# Light brightness data near sea turtle nests as measured from the horizon and zenith using a Sky Quality Meter

**DOI:** 10.1016/j.dib.2022.108430

**Published:** 2022-07-01

**Authors:** Shigetomo Hirama, Andrea Sylvia, Tonya Long, Robbin Trindell, Blair Witherington

**Affiliations:** a Florida Fish and Wildlife Conservation Commission, Fish and Wildlife Research Institute, 1105 Southwest Williston Road, Gainesville, FL 32601, USA; b Florida Fish and Wildlife Conservation Commission, Fish and Wildlife Research Institute, Tequesta Field Laboratory, 19100 Southeast Federal Highway, Tequesta, FL 33469, USA; c Florida Fish and Wildlife Conservation Commission, Imperiled Species Management, 1875 Orange Avenue East, Tallahassee, FL 32311, USA; d Inwater Research Group Inc., 4160 NE Hyline Dr., Jensen Beach, FL 34957, USA

**Keywords:** SQM, Light pollution, Ambient light, Sky glow, Photometry, Sea turtle, Hatchling orientation, Hatchling disorientation

## Abstract

A Sky Quality Meter (SQM) is a photometer designed to measure night sky brightness. SQMs have been used to interpret light fields for various purposes, including studies of relationships between directional light brightness and sea turtle hatchling orientation. This article reports SQM data collected on a sea turtle nesting beach at Delray Beach, Florida (USA) on 17 August 2020. Three hundred-and-sixty light brightness data were collected by eight consecutive readings in each of the four horizontal directions and the zenith at nine sites. The data also include landward photographic images captured at the nine light-measurement sites. The dataset supports the usefulness of the instrument for biological light-field measurements and refute blanket criticisms on inconsistent measurements that would justify discarding initial readings and averaging over a time series.

## Specifications Table

**Table:** 

Subject	Pollution
Specific subject area	Light brightness data that measure light pollution relative to sea turtle nest sites and seaward hatchling orientation.
Type of data	Table, Image, Figure.
How data were acquired	We used a Sky Quality Meter (Unihedron, model: SQM-L), DSLR camera (Nikon D5300 with Nikkor 18–55 mm f/3.5 G lens, ISO-1600, exposure time 20 s). Other equipment included a tripod with a cross-shape platform, and a level. The statistical program used was R (version 3.3.1).
Data format	Raw, Analyzed, Filtered.
Description of data collection	The photometric data were collected at Delray Beach, Florida, USA on 17 August 2020 (one day before a new moon night), between 21:30 and 23:05 DST (UTC-4 hrs). The moonrise on the night was at 04:30, 18 August 2020.
Data accessibility	Florida Fish and Wildlife Conservation Commission, Fish and Wildlife Research Institute,Gainesville, Florida, USA. Repository name: Light brightness data near sea turtle nests as measured from the horizon and zenith using a Sky Quality Meter. Raw data repository at:https://f50006a.eos-intl.net/F50006A/OPAC/Details/Record.aspx?BibCode=5543762The link to the raw data is available at the lower part of the above website.

## Value of the Data

• Light brightness measurements from an SQM directed toward the horizon on a sea turtle nesting beach have been described as untrustworthy and non-repeatable because of high variance between measurements [1,2]. The present data are important because of the contribution to a test of this hypothesis.
• Researchers who wish to understand the relationship between light pollution on sea turtle nesting beaches and the behaviors of nesting adults and hatchlings benefit from an assessment of these data. If the performance of SQM measurements is suitable, researchers have justification in selecting a relatively low-cost photometer to conduct their studies.
• The data showed no statistical differences in inter-site variation between horizontal SQM measurements and those measured at zenith; both the horizontal and zenith measurements showed similar variations. The data that were collected from the horizon could be used for comparisons to similar tests at other nesting beach locations.
• The data can be used to understand light-field contributions from artificial lighting, within the spectrum of 480 to 600 nm. The data are most valuable in association with standardized night-time photos, additional photometry within the spectrum of 480–340 nm, and field arena assay experiments using light-naïve hatchlings.

## 1 Data Description

**Fig. 1:**
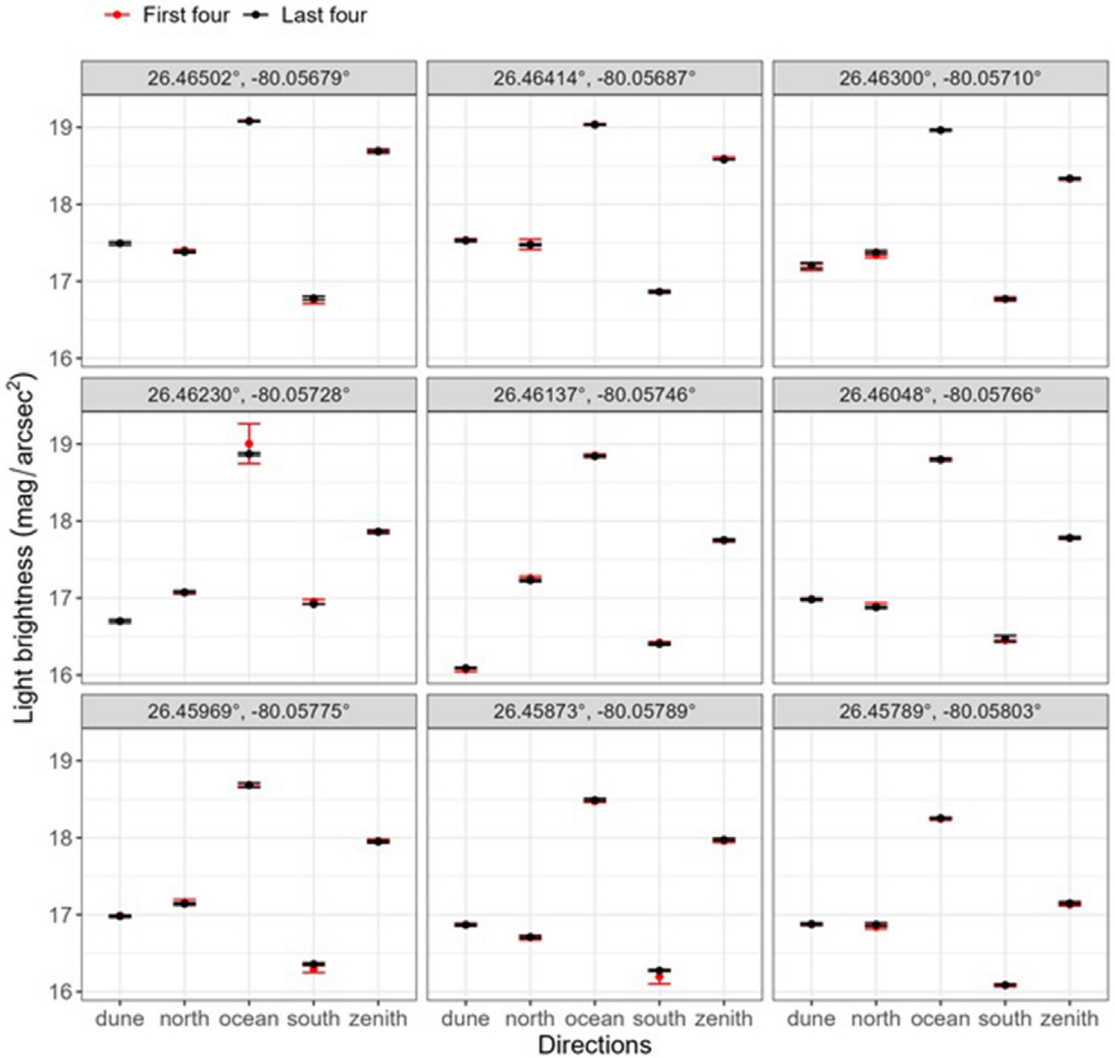
SQM-L light measurements on Delray Beach for the first four (1st–4th; red, *n* = 180) and the last four (5th–8th; black, *n* = 180) readings, across sites (panels) and directions (x-axis). Points represent the mean value of the four readings and lines represent the standard deviation of the four light brightness readings. Larger magnitude per arcsecond squared-values indicate lower light brightness, darker conditions. North and south directions are parallel to the shoreline.

**Fig. 2:**
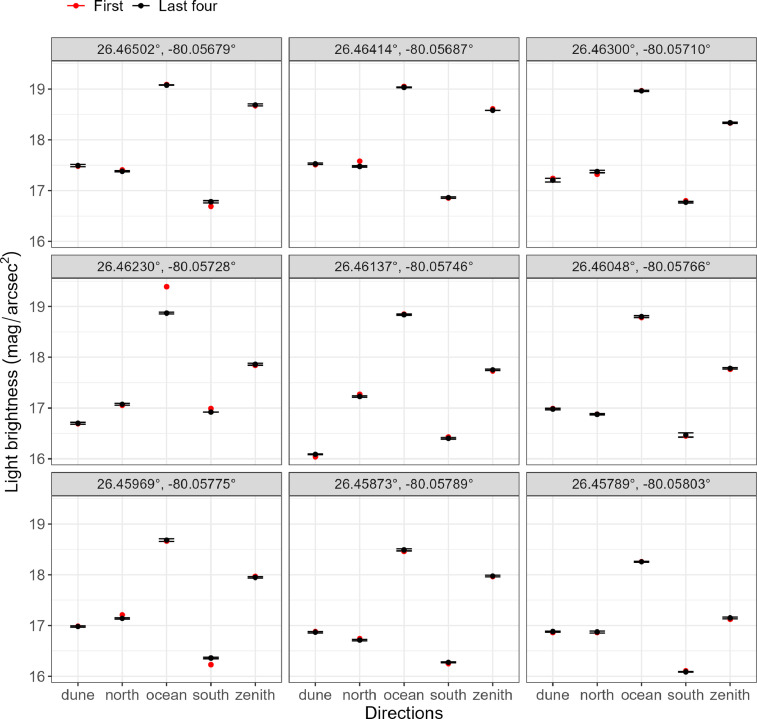
Light brightnesses measured on Delray Beach first (red, *n* = 45) and last four (5th–8th; black, *n* = 180) readings, across beaches (panels) and directions (x-axis). Points represent the mean value of the four readings and lines represent the standard deviation of the four light brightness readings. Larger magnitude per arcsecond squared-values indicate lower light brightness, darker conditions. North and south directions are parallel to the shoreline.

The SQM-L measures field brightness in star magnitudes per square arc second (mag/arcsec^2^), such that smaller readings indicate a brighter light field. We present data for light brightness measured by an SQM-L aimed in four azimuthal directions plus zenith, totaling 360 light readings [8 readings (raw data column name “order”) per direction, 5 directions (raw data column name “direction”) per site, 9 sites (raw data column names “lat” and “lon” combined)]. Across all sites and directions, all measurements (*n* = 360) other than at a single site were self-consistent (Table 2), defined as exceeding a maximum of approximately 0.2 mag/arcsec^2^ [1]. The largest difference in readings was seen between the first and fourth measurements and fifth through eight measurements at site 26.46230°, −80.05728, ocean direction (Figs. 1 and 2).

### 1.1 Comparing the First–Fourth with Fifth–Eighth Light Readings in all Directions

Plots of the raw data show a strong overlap between the readings of the first (1–4) and second (5–8) sets of measurements (Fig. 1). The average range of light brightness readings in the first four samples was 0.06 mag/arcsec^2^, which was slightly larger than that of the second four samples (0.03 mag/arcsec^2^) (Fig. 1). Although discarding the first 3–4 SQM readings of light brightness is recommended [1], our tests indicated no statistically significant differences between the first four readings and the second four readings (*V* = 443, *p* = 0.72).

### 1.2 Comparing the First with Fifth–Eighth Light Readings in all Directions

Data split by the first reading and fifth-through-eighth readings also resulted in no statistically significant differences between samples (*V* = 511.5, *p* = 0.95, Fig. 2). The median difference between the pooled scenarios was 0.04 mag/arcsec^2^ (Table 1). The range of data was larger for the single readings at 3.35 compared to the averaged readings at 2.99 (Table 1). Plots of the raw data showed similarities between light brightness levels with few occurrences of relatively large differences. For example, in the ocean direction at 26.46230°, −80.05728°, the first reading (19.39 mag/arcsec^2^) was 0.51 mag/arcsec^2^ larger than the mean of the 5th through 8th readings (18.88 mag/arcsec^2^; Fig. 2).

**Table 1:** Light-measurements repeated eight times in each of five directions at nine sites, totaling 360 readings. The columns 1st, 1st–4th, and 5th–8th, show statistical characteristics of the light data that were measured as the first, first to fourth, and fifth to eighth readings, respectively. The units of values are in magnitude per arcsecond squared (mag/arcsec^2^). Sd is the standard deviation.

Readings	1st (*n* = 45)	1st–4th (*n* = 180)	5th–8th (*n* = 180)
Min – Max	16.04–19.39	16.07–19.09	16.09–19.08
Mean (sd)	17.50 (0.91)	17.49 (0.89)	17.49 (0.88)
Median	17.27	17.26	17.23

**Table 2:** Summary statistics of 1st–4th light measurements in five directions (dune, north, ocean, south, zenith) at nine sites. North and south directions are parallel to the shoreline. The summary includes median, mean with standard deviation (sd), and maximum – minimum with difference for each site and direction. The units of values are in magnitude per arcsecond squared (mag/arcsec^2^). Sd is the standard deviation. The geographic coordinates are in decimal degrees.

Site	Direction	Median	Mean (sd)	Max-Min (difference)
26.46502°, −80.05679°	dune	17.49	17.49 (0.019)	17.52–17.48 (0.04)
26.46502°, −80.05679°	north	17.41	17.40 (0.010)	17.41–17.39 (0.02)
26.46502°, −80.05679°	ocean	19.09	19.09 (0.006)	19.09–19.08 (0.01)
26.46502°, −80.05679°	south	16.78	16.76 (0.047)	16.79–16.69 (0.10)
26.46502°, −80.05679°	zenith	18.71	18.70 (0.019)	18.71–18.67 (0.04)
26.46414°, −80.05687°	dune	17.54	17.54 (0.017)	17.55–17.51 (0.04)
26.46414°, −80.05687°	north	17.45	17.48 (0.067)	17.58–17.44 (0.14)
26.46414°, −80.05687°	ocean	19.04	19.04 (0.008)	19.05–19.03 (0.02)
26.46414°, −80.05687°	south	16.87	16.87 (0.017)	16.88–16.85 (0.03)
26.46414°, −80.05687°	zenith	18.60	18.60 (0.017)	18.61–18.58 (0.03)
26.46300°, −80.05710°	dune	17.17	17.18 (0.040)	17.24–17.15 (0.09)
26.46300°, −80.05710°	north	17.33	17.34 (0.029)	17.38–17.32 (0.06)
26.46300°, −80.05710°	ocean	18.96	18.96 (0.005)	18.97–18.96 (0.01)
26.46300°, −80.05710°	south	16.77	16.77 (0.025)	16.80–16.74 (0.06)
26.46300°, −80.05710°	zenith	18.33	18.33 (0.016)	18.35–18.31 (0.04)
26.46230°, −80.05728°	dune	16.69	16.70 (0.020)	16.73–16.69 (0.04)
26.46230°, −80.05728°	north	17.07	17.07 (0.013)	17.08–17.05 (0.03)
26.46230°, −80.05728°	ocean	18.88	19.00 (0.259)	19.39–18.86 (0.53)
26.46230°, −80.05728°	south	16.95	16.96 (0.026)	16.99–16.93 (0.06)
26.46230°, −80.05728°	zenith	17.85	17.86 (0.017)	17.88–17.84 (0.04)
26.46137°, −80.05746°	dune	16.07	16.07 (0.025)	16.10–16.04 (0.06)
26.46137°, −80.05746°	north	17.27	17.26 (0.022)	17.28–17.23 (0.05)
26.46137°, −80.05746°	ocean	18.86	18.85 (0.017)	18.87–18.83 (0.04)
26.46137°, −80.05746°	south	16.42	16.42 (0.012)	16.43–16.41 (0.02)
26.46137°, −80.05746°	zenith	17.75	17.75 (0.017)	17.76–17.73 (0.03)
26.46048°, −80.05766°	dune	16.99	16.99 (0.013)	17.00–16.97 (0.03)
26.46048°, −80.05766°	north	16.89	16.90 (0.032)	16.95–16.88 (0.07)
26.46048°, −80.05766°	ocean	18.79	18.79 (0.013)	18.81–18.78 (0.03)
26.46048°, −80.05766°	south	16.45	16.45 (0.000)	16.45–16.45 (0.00)
26.46048°, −80.05766°	zenith	17.78	17.78 (0.015)	17.79–17.76 (0.03)
26.45969°, −80.05775°	dune	16.99	16.99 (0.000)	16.99–16.99 (0.00)
26.45969°, −80.05775°	north	17.15	17.17 (0.030)	17.21–17.15 (0.06)
26.45969°, −80.05775°	ocean	18.70	18.69 (0.022)	18.71–18.66 (0.05)
26.45969°, −80.05775°	south	16.31	16.30 (0.045)	16.33–16.23 (0.10)
26.45969°, −80.05775°	zenith	17.97	17.96 (0.020)	17.97–17.93 (0.04)
26.45873°, −80.05789°	dune	16.87	16.87 (0.015)	16.89–16.86 (0.03)
26.45873°, −80.05789°	north	16.70	16.70 (0.029)	16.74–16.68 (0.06)
26.45873°, −80.05789°	ocean	18.48	18.47 (0.010)	18.48–18.46 (0.02)
26.45873°, −80.05789°	south	16.23	16.20 (0.093)	16.26–16.06 (0.20)
26.45873°, −80.05789°	zenith	17.96	17.96 (0.017)	17.98–17.94 (0.04)
26.45789°, −80.05803°	dune	16.87	16.87 (0.013)	16.89–16.86 (0.03)
26.45789°, −80.05803°	north	16.85	16.84 (0.022)	16.86–16.81 (0.05)
26.45789°, −80.05803°	ocean	18.25	18.25 (0.017)	18.26–18.23 (0.03)
26.45789°, −80.05803°	south	16.08	16.09 (0.017)	16.11–16.07 (0.04)
26.45789°, −80.05803°	zenith	17.13	17.13 (0.014)	17.15–17.12 (0.03)

### 1.3 Comparing Zenith with Horizontal Direction using the Fifth–Eighth Light Readings

**Fig. 3:**
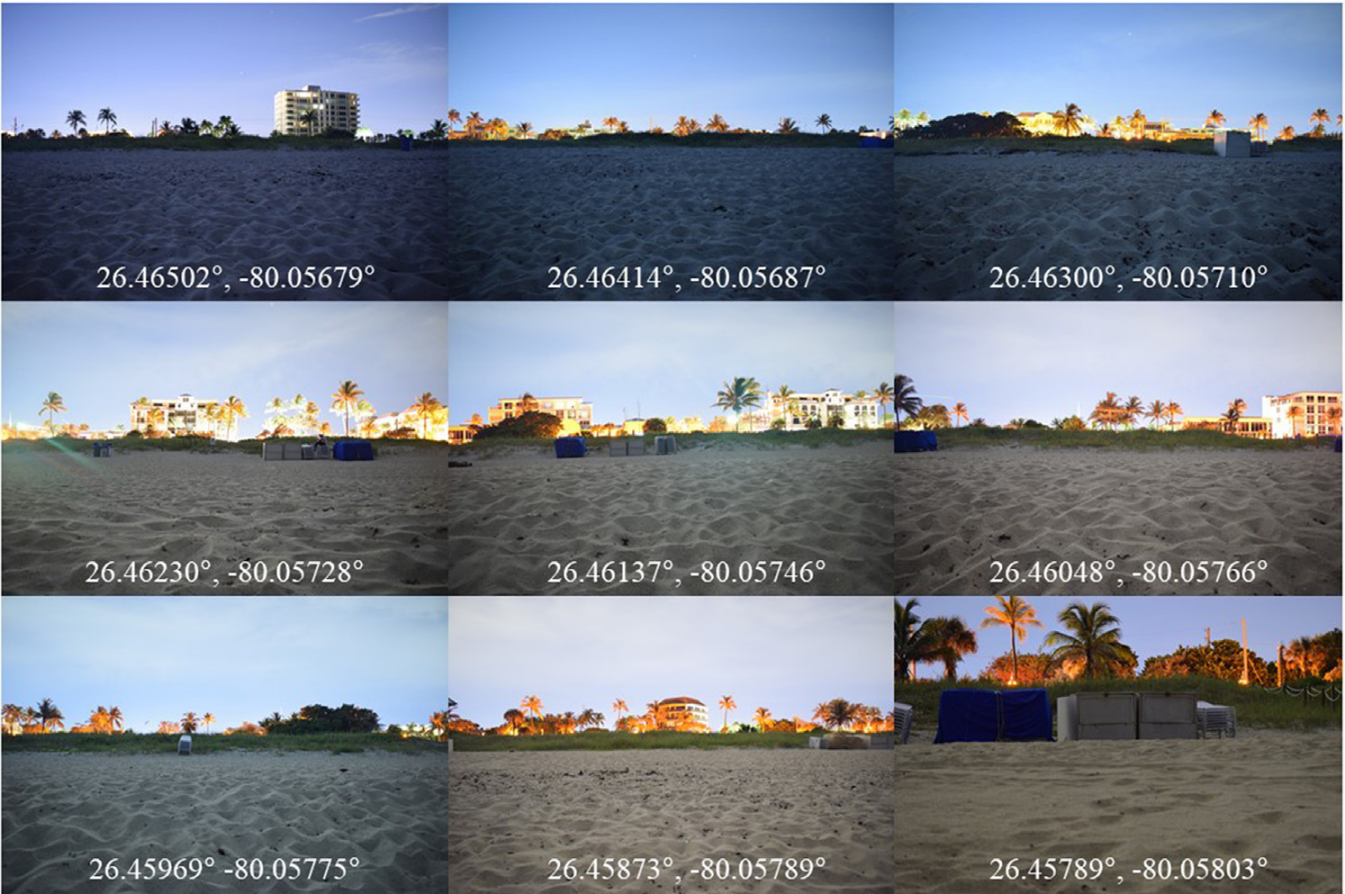
Photographic images that were captured immediately after measuring light brightness at the nine sites. The geographic coordinates of the sites and times (Eastern Standard Time) of photographing were: 26.46502°, −80.05679°, 23:05; 26.46414°, −80.05687°, 22:50; 26.46300°, −80.05710°, 22:38; 26.46230°, −80.05728°, 22:26; 26.46137°, −80.05746°, 22:19; 26.46048°, −80.05766°, 22:06; 26.45969°, −80.05775°, 21:54; 26.45873°, −80.05789°, 21:31; 26.45789°, −80.05803°, 21:12, with different zoom percentage than other sites.

The variances in SQM measurements at the zenith and toward horizontal directions were similar across sites. The photographic images (Fig. 3) visually and subjectively confirmed the relative light-intensity values collected by the SQM-L aimed landward (Table 3, Figs. 1 and 2).

**Table 3:** Summary statistics of 5th–8th light measurements (mag/arcsec^2^) in five directions (dune, north, ocean, south, zenith) at nine sites . North and south directions are parallel to the shoreline. The summary includes median, mean with standard deviation (sd), and maximum – minimum with difference for each site and direction. The units of values are in magnitude per arcsecond squared (mag/arcsec^2^). Sd is the standard deviation.

Site	Direction	Median	Mean (sd)	Max-Min (difference)
26.46502°, −80.05679°	dune	17.50	17.49 (0.021)	17.51–17.47 (0.04)
26.46502°, −80.05679°	north	17.38	17.38 (0.012)	17.39–17.37 (0.02)
26.46502°, −80.05679°	ocean	19.08	19.08 (0.005)	19.08–19.07 (0.01)
26.46502°, −80.05679°	south	16.78	16.78 (0.022)	16.81–16.76 (0.05)
26.46502°, −80.05679°	zenith	18.69	18.69 (0.021)	18.71–18.67 (0.04)
26.46414°, −80.05687°	dune	17.53	17.53 (0.015)	17.54–17.51 (0.03)
26.46414°, −80.05687°	north	17.48	17.48 (0.013)	17.49–17.46 (0.03)
26.46414°, −80.05687°	ocean	19.03	19.03 (0.005)	19.04–19.03 (0.01)
26.46414°, −80.05687°	south	16.86	16.86 (0.015)	16.88–16.85 (0.03)
26.46414°, −80.05687°	zenith	18.58	18.58 (0.000)	18.58–18.58 (0.00)
26.46300°, −80.05710°	dune	17.21	17.21 (0.035)	17.24–17.17 (0.07)
26.46300°, −80.05710°	north	17.39	17.38 (0.024)	17.39–17.34 (0.05)
26.46300°, −80.05710°	ocean	18.96	18.96 (0.013)	18.98–18.95 (0.03)
26.46300°, −80.05710°	south	16.77	16.77 (0.014)	16.79–16.76 (0.03)
26.46300°, −80.05710°	zenith	18.34	18.34 (0.010)	18.34–18.32 (0.02)
26.46230°, −80.05728°	dune	16.70	16.70 (0.018)	16.72–16.68 (0.04)
26.46230°, −80.05728°	north	17.08	17.08 (0.019)	17.09–17.05 (0.04)
26.46230°, −80.05728°	ocean	18.87	18.87 (0.018)	18.89–18.85 (0.04)
26.46230°, −80.05728°	south	16.92	16.92 (0.000)	16.92–16.92 (0.00)
26.46230°, −80.05728°	zenith	17.86	17.86 (0.019)	17.89–17.85 (0.04)
26.46137°, −80.05746°	dune	16.09	16.09 (0.008)	16.10–16.08 (0.02)
26.46137°, −80.05746°	north	17.23	17.23 (0.015)	17.24–17.21 (0.03)
26.46137°, −80.05746°	ocean	18.84	18.84 (0.012)	18.85–18.83 (0.02)
26.46137°, −80.05746°	south	16.40	16.40 (0.015)	16.42–16.39 (0.03)
26.46137°, −80.05746°	zenith	17.76	17.75 (0.015)	17.76–17.73 (0.03)
26.46048°, −80.05766°	dune	16.98	16.98 (0.014)	17.00–16.97 (0.03)
26.46048°, −80.05766°	north	16.88	16.88 (0.013)	16.89–16.86 (0.03)
26.46048°, −80.05766°	ocean	18.80	18.80 (0.018)	18.82–18.78 (0.04)
26.46048°, −80.05766°	south	16.48	16.47 (0.042)	16.51–16.42 (0.09)
26.46048°, −80.05766°	zenith	17.79	17.78 (0.015)	17.79–17.76 (0.03)
26.45969°, −80.05775°	dune	16.99	16.98 (0.014)	16.99–16.96 (0.03)
26.45969°, −80.05775°	north	17.15	17.14 (0.014)	17.15–17.12 (0.03)
26.45969°, −80.05775°	ocean	18.69	18.68 (0.028)	18.71–18.65 (0.06)
26.45969°, −80.05775°	south	16.36	16.36 (0.014)	16.38–16.35 (0.03)
26.45969°, −80.05775°	zenith	17.95	17.95 (0.015)	17.96–17.93 (0.03)
26.45873°, −80.05789°	dune	16.86	16.87 (0.015)	16.89–16.86 (0.03)
26.45873°, −80.05789°	north	16.72	16.71 (0.015)	16.72–16.69 (0.03)
26.45873°, −80.05789°	ocean	18.48	18.49 (0.020)	18.52–18.48 (0.04)
26.45873°, −80.05789°	south	16.27	16.28 (0.010)	16.29–16.27 (0.02)
26.45873°, −80.05789°	zenith	17.98	17.98 (0.017)	17.99–17.95 (0.04)
26.45789°, −80.05803°	dune	16.88	16.88 (0.012)	16.89–16.87 (0.02)
26.45789°, −80.05803°	north	16.87	16.87 (0.021)	16.90–16.85 (0.05)
26.45789°, −80.05803°	ocean	18.26	18.26 (0.010)	18.26–18.24 (0.02)
26.45789°, −80.05803°	south	16.09	16.09 (0.005)	16.09–16.08 (0.01)
26.45789°, −80.05803°	zenith	17.15	17.15 (0.018)	17.17–17.13 (0.04)

## 2 Experimental Design, Materials and Methods

### 2.1 Study Site, Equipment, and Measuring Light

**Fig. 4:**
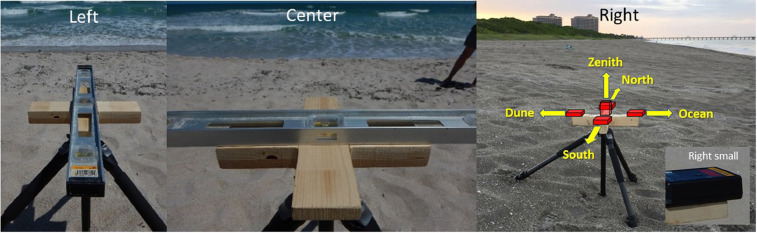
Images of the cross-shape platform attached to the camera tripod (Left, Center, Right). The red boxes and yellow arrows (Right) show measuring positions of the SQM-L and the aiming directions of the sensor. The SQM-L was placed on the platform in such way that its edge avoided overlapping with the platform (Right small).

On 17 August 2020, we measured light brightness and took digital photographs at sea turtle nesting areas. These data were collected at nine sites about 100 m apart along approximately 800 m in Delray Beach, Florida, USA (Fig. 3). The data were collected during moonless portions of the night from 21:12 to 23:05 (US Eastern Daylight Time, UTC-4 h). We measured light intensities toward the horizon parallel to the shoreline both north and south, toward the horizon both landward and seaward perpendicular to the shore-parallel directions, and toward the zenith. This was done from a cross-shaped platform attached to a camera tripod (Fig. 4). The height of the ocular part of the SQM (Unihedron, model: SQM-L) above the surface of the sand at each site was 50 cm for the horizon measurements and 59 cm for the zenith measurement. On the night of the work, we observed occasional passing clouds (Fig. 3).

After leveling the two axes of the measuring platform at each site (Fig. 4 Left and Center), the light intensities were measured in five directions, two directions parallel to the shoreline (north– and southward), duneward, oceanward, and zenith (Fig. 4 Right). At each of the nine sites, we measured light brightness. We repeated the measurement eight times in each direction, measuring 40 readings per site, totalling 360 readings in nine sites. To avoid possible reflection of light, the SQM-L was placed on the platform in such a way that its sensor did not overlap the edge of the platform (Fig. 4 Right small). The person who operated the SQM-L pushed its button with a crouched-down body position. The observers wore black clothes to minimize reflection of light. Immediately after collecting the light brightness data and before moving to the next site, we photographed the landward placing a DLSR camera (Nikon D5300, lens: NIKKOR 18–55 mm f/3.5 G, ISO–1600, exposure time 20 s) on the light measuring platform. To eliminate movement of the camera while pressing its shutter button, we set its timer function to photograph. Unintentionally, we captured one of the photographic images in a different zoom focal length (Fig. 3 26.45789°, −80.05803°).

### 2.2 Data Management and Analyses

RStudio (Version 1.2.5019) was used for all data processing described above. A Wilcoxon signed-rank test with a significance level at α = 0.05 was used to test for differences between datasets because the data followed a non-normal distribution.

## Ethical Statement

All work was conducted in accordance with a Section 6 Cooperative Agreement between FWC and the U.S. Fish and Wildlife Service or under an FWC permit issued in accordance with Florida Statute 379.243(1).

## CRediT authorship contribution statement

**Shigetomo Hirama:** Conceptualization, Methodology, Data curation, Writing – original draft, Writing – review & editing, Supervision. **Andrea Sylvia:** Visualization, Writing – review & editing. **Tonya Long:** Data curation, Writing – review & editing. **Robbin Trindell:** Data curation, Writing – review & editing. **Blair Witherington:** Conceptualization, Methodology, Writing – review & editing.

## Declaration of Competing Interest

The authors declare that they have no known competing financial interests or personal relationships which have or could be perceived to have influenced the work reported in this article.
